# Aggressive and Refractory Attack of AQP4-IgG-Positive Neuromyelitis Optica Spectrum Disorder Treated With Ravulizumab: A Case Report

**DOI:** 10.7759/cureus.82650

**Published:** 2025-04-20

**Authors:** José M Valdés, Patricia Orellana, Marianella Hernandez, Jorge Barahona, Lorna Galleguillos

**Affiliations:** 1 Neurology and Psychiatry, Clinica Alemana, Santiago, CHL; 2 Radiology, Hospital Clínico de la Universidad de Chile, Santiago, CHL; 3 Neuroradiology, Clinica Alemana, Santiago, CHL

**Keywords:** acute attack, aquaporin-4, disease-modifying therapy, neuromyelitis optica spectrum disorder (nmosd), ravulizumab

## Abstract

Neuromyelitis optica spectrum disorder (NMOSD) is a severe autoimmune astrocytopathy mediated by aquaporin-4-immunoglobulin G (AQP4-IgG) antibodies, leading to complement-mediated neural injury. While ravulizumab, a long-acting C5 inhibitor, is approved for relapse prevention in AQP4-IgG-positive NMOSD, its role in acute attacks remains unestablished. We report a 58-year-old woman with a highly relapsing course of AQP4-IgG-positive NMOSD who developed a fulminant attack with bilateral corticospinal tract involvement, resulting in asymmetrical tetraparesis. Despite high-dose intravenous methylprednisolone and plasma exchange (PLEX), neurological deterioration progressed. Ravulizumab (2,700 mg) was administered emergently following meningococcal prophylaxis. After treatment, neurological decline ceased, allowing gradual motor recovery and rehabilitation. At discharge, the patient showed improved strength and functional capacity, although significant disability persisted. This case suggests that ravulizumab may provide clinical stabilization in refractory NMOSD attacks unresponsive to conventional therapy. The temporal association between ravulizumab administration and the halt in clinical progression suggests a possible therapeutic effect; however, delayed responses to steroids and PLEX, along with the confounding use of intravenous Ig, preclude definitive attribution. While the use of complement inhibitors has been well established in relapse prevention, evidence for their use in acute NMOSD exacerbations remains limited. This report highlights the need for controlled studies to evaluate the efficacy, safety, and timing of ravulizumab in the management of acute, treatment-resistant NMOSD relapses.

## Introduction

Ravulizumab has emerged as a significant therapeutic option for the treatment of patients with neuromyelitis optica spectrum disorder (NMOSD) who are positive for aquaporin-4 (AQP4) antibodies. Its clinical utility is largely attributed to its mechanism as a terminal complement inhibitor, addressing the complement-mediated pathophysiology underlying NMOSD [1].

Recent evidence indicates that ravulizumab can provide immediate and sustained suppression of serum complement component C5, thereby preventing the damage caused by NMOSD attacks [2, 3]. The CHAMPION-NMOSD trial demonstrated a marked reduction in relapse risk following ravulizumab treatment, highlighting its potential as a long-acting therapeutic option for NMOSD [4]. The pharmacodynamics of ravulizumab reveal that it achieves complete C5 inhibition shortly after administration, which is critical in managing acute inflammatory responses during NMOSD attacks [5,6].

Although the primary clinical indication for ravulizumab remains relapse prevention, isolated case reports have suggested a potential role for terminal complement inhibition during acute NMOSD attacks, where rapid modulation of inflammatory cascades may offer symptomatic benefit [7,8]. However, this application remains investigational and is not currently supported by controlled clinical trial data. Notably, similar early therapeutic effects have been observed in other complement-mediated disorders, such as generalized myasthenia gravis, further supporting the plausibility of rapid clinical improvement in acute immunologic exacerbations [5,9,10].

## Case presentation

A 58-year-old female patient was diagnosed with NMOSD seropositive for AQP4 antibodies in 2009. Retrospectively, her initial symptoms began in 2007, suggesting a possible area postrema syndrome with a negative MRI, followed later that year by post-vaccination transverse myelitis. Over the following years, she experienced multiple recurrent episodes of myelitis and optic neuritis, exhibiting a particularly refractory disease course. Rituximab therapy was initiated; however, poor adherence resulted in more than 15 relapses. Surprisingly, her Expanded Disability Status Scale (EDSS) score remained at three.

On February 14, 2025, the patient awoke with spontaneous vertiginous sensations, which improved upon lying laterally. By the following day, she developed right-sided lateropulsion and subacute dysarthria. Notably, nausea, vomiting, hiccups, and visual field deficits were absent.

**Figure 1:**
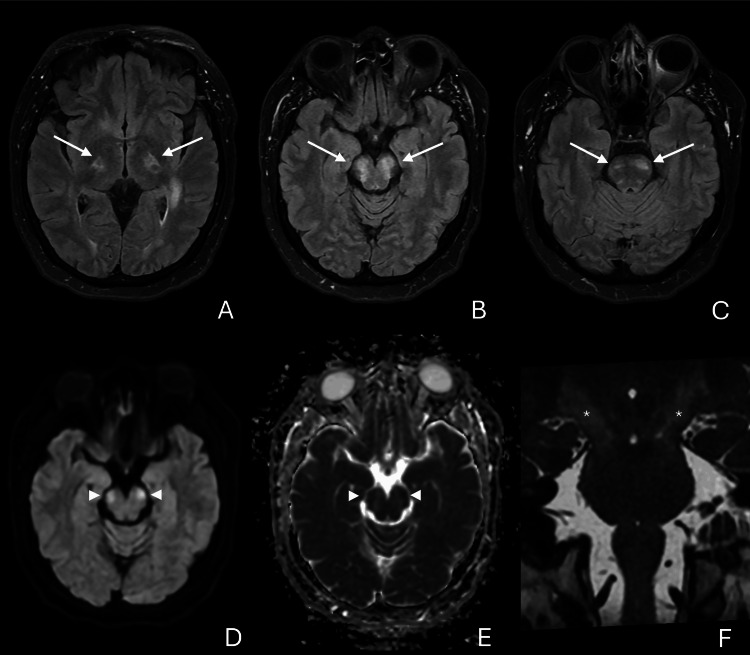
Brain MRI findings (A–C) Axial T2-weighted fluid-attenuated inversion recovery (FLAIR) images demonstrate bilateral hyperintensities along the corticospinal tracts (white arrows), indicative of tract-specific involvement; (D–E) Diffusion-weighted imaging (DWI) shows areas of restricted diffusion (arrowheads), with corresponding hypointensity on the apparent diffusion coefficient (ADC) map, consistent with cytotoxic edema in acute lesions; (F) Coronal T2-weighted sequence further delineates tract involvement, showing longitudinal hyperintensity extending from the posterior limb of the internal capsule through the cerebral peduncles to the pontine base (asterisks), tracking the full course of the corticospinal tracts.

Upon evaluation in the emergency department on February 16, vascular protocol imaging ruled out ischemic and hemorrhagic stroke. Due to patient agitation, initial MRI completion was impractical. Nevertheless, empirical intravenous methylprednisolone (1 g daily for five days) was administered (day 1). On day 2, a brain MRI performed under anesthesia revealed extensive acute bilateral corticospinal tract involvement (Figure 1), while the patient developed asymmetrical tetraparesis.

Cerebrospinal fluid (CSF) analysis showed mild pleocytosis (7.5 leukocytes/mm³, reference range (RR): 0-5 leukocytes/mm³), a slightly elevated protein level (47.8 mg/dL, RR: 15-45 mg/dL) with preserved glucose, and positivity for aquaporin-4-immunoglobulin G (AQP4-IgG). The CSF autoimmune encephalitis panel was negative. Additionally, a comprehensive rheumatological evaluation ruled out any concurrent systemic autoimmune diseases, and no viral or bacterial pathogens were detected.

Given the progression of neurological deficits, plasma exchange (PLEX) was initiated on day 3 (1.5 plasma volumes per day for five days). However, the patient continued to deteriorate, developing upgaze palsy, mild dysphagia, and tetraparesis against gravity, with an EDSS of 9.0 by day 8.

Due to the severity and refractoriness of the attack, emergency treatment with ravulizumab (2,700 mg) was initiated on day 12, following meningococcal vaccination and prophylactic ciprofloxacin. Given PLEX-induced hypogammaglobulinemia, intravenous immunoglobulin (IVIG) replacement therapy was also administered.

After ravulizumab treatment, progression ceased, allowing rehabilitation to advance. At discharge on day 19, the patient was alert and oriented, with fluent but mildly dysarthric speech. Optic nerve atrophy was evident bilaterally, with bilateral relative afferent pupillary defect and no ophthalmoparesis or mild right-sided central facial paresis. Right-sided hemiparesis (Medical Research Council (MRC) grade 4-), with less marked left-side weakness (MRC 4+), hyper-reflexia (3/5) in all extremities, and bilateral extensor plantar response. Reduced vibration sense in the lower extremities bilaterally. Ataxia in the context of tetraparesis was evident. The patient required dual assistance for ambulation, with a walking range of less than 20 meters. Her EDSS was 7.0, and her modified Rankin Scale score was four. No headache, fever, or meningeal signs developed during hospitalization.

## Discussion

AQP-4 positive NMOSD is a severe autoimmune astrocytopathy characterized by recurrent episodes of demyelination, primarily targeting the optic nerves and spinal cord. AQP4-IgG facilitates complement-mediated astrocyte injury, triggering inflammatory cascades via membrane attack complex (MAC) activation, astrocyte destruction, and subsequent recruitment of inflammatory cells that contribute to extensive neural damage and disability progression.

The management of acute NMOSD attacks traditionally involves high-dose corticosteroids and PLEX [1,11]. Ravulizumab, an FDA-approved modifying therapy [12], achieves rapid and sustained inhibition of C5 cleavage, preventing MAC formation and downstream inflammatory damage [4,13]. Although its clinical utility has been demonstrated in preventing relapses, evidence regarding its use during acute exacerbations is limited.

The patient had a refractory disease course, experiencing over 15 relapses despite rituximab therapy. The reported acute episode presented with bilateral corticospinal tract involvement and progressive tetraparesis, raising concerns for severe morbidity. Given the failure of high-dose corticosteroids and PLEX to arrest clinical progression, ravulizumab was administered emergently as a rescue therapy.

Despite the patient’s severe presentation, ravulizumab administration led to cessation of neurological deterioration. However, the delayed effects of corticosteroids and PLEX, which may take several days to exert their full therapeutic impact [1,14-17], could have also contributed to the patient’s recovery, making it difficult to isolate ravulizumab’s direct role. The confounding influence of concurrent therapies, including corticosteroids, PLEX, and IVIG, remains a key limitation, highlighting the need for further investigation into the synergistic or independent effects of complement inhibition in NMOSD exacerbations, as existing data primarily focus on annualized relapse reduction rather than acute exacerbation resolution.

Despite these limitations, this case adds meaningful real-world evidence supporting the feasibility and safety of ravulizumab in the acute setting, supporting its potential as a rescue therapy [13,18,19]. The novel application of complement inhibition in this context may be particularly relevant in cases with high disease burden and poor response to B-cell depletion or conventional immunosuppression.

Further research is necessary to delineate the therapeutic role of complement inhibitors in acute NMOSD attacks. Prospective studies should aim to evaluate clinical efficacy, optimal timing of administration, and long-term outcomes, particularly in comparison to traditional escalation strategies. Until such data become available, our case underscores the importance of mechanism-targeted therapies in severe NMOSD and supports consideration of ravulizumab in select acute scenarios where rapid complement inhibition may mitigate irreversible damage.

## Conclusions

This case underscores the potential role of ravulizumab as an adjunctive therapy in the acute management of NMOSD, particularly in patients with treatment-refractory disease. While the observed clinical stabilization following ravulizumab administration is encouraging, these findings remain preliminary and should be interpreted with caution given the confounding influence of concurrent therapies. Nevertheless, this real-world observation contributes to the evolving therapeutic landscape of NMOSD and highlights the need for prospective studies to clarify the efficacy, optimal timing, and long-term outcomes of complement inhibition during acute exacerbations.
